# Effect of different types of oil intake on the blood index and the intestinal flora of rats

**DOI:** 10.1186/s13568-022-01387-w

**Published:** 2022-05-05

**Authors:** Yan Xu, Wenzheng Zhu, Qingfeng Ge, Xiaoyan Zhou

**Affiliations:** 1 School of Food Science and Engineering, Yangzhou University, No.196 Huayang West Road, Hanjiang District, Yangzhou City, 225127 Jiangsu Province China; 2 College of Animal Science and Technology, Yangzhou University, No.196 Huayang West Road, Hanjiang District, Yangzhou City, 225127 Jiangsu Province China; 3 Key Laboratory of Chinese Cuisine Intangible Cultural Heritage Technology Inheritance, Ministry of Culture and Tourism, Yangzhou University, Yangzhou, 225127 China

**Keywords:** Dietary lipids, Fatty acids, Blood lipids, 16S rRNA sequencing, Gut microbiota

## Abstract

Dietary fat is an important part of human diet and has a close relationship with human health. However, it is still unclear how gut microbiota in adolescent responds to dietary fats at a normal dose. In this study, fat-free group (BC) was used as blank control group, we explored blood index and gut microbiota structure in growing rat(aged 1 months) after feeding a normal dose of 16.9% stewed lard(SL), refined lard(RL), fish oil(FO) and soybean oil(SO) for 6 weeks, respectively. The results showed that compared with RL group, SL group showed reduced fasting blood sugar and blood lipid levels and improved nutrient absorption capacity of the intestine. The blood indexes of glucose (Glu), total cholesterol (TC) and total triglyceride (TG) in FO treatment group were relatively low. The abundance of *Bacteroidetes* in the BC group decreased, and the abundance of *Firmicutes* increased. The *Firmicutes/Bacteroidetes* ratio of the FO group was relatively low, and the *Firmicutes/Bacteroidetes* ratio of the SL group and the SO group was lower than that of the RL group. The abundance of *Bacteroidaceae* in the SL group was increased. Research results showed that fat-free diets will increase the risk of obesity to a certain extent; compared with refined lard, stewed lard, soybean oil and fish oil can reduce the risk of obesity to a certain extent. The present study could find that the addition and types of dietary fat will affect the abundance and diversity of rat intestinal flora, and provide some information for nutritional evaluation about these dietary lipids.

**Supplementary Information:**

The online version contains supplementary material available at 10.1186/s13568-022-01387-w.

## Introduction

Dietary fat is an important part of human diet, and its nutritional value depends on a large extent on the composition of fatty acids, which is closely related to human health (Roche et al. 2014). Different dietary fats have different compositions of fatty acids, lard and SO are commonly used edible oils for Chinese residents, and FO is the most studied health oil: SL is obtained by simmering pork belly in low heat for a long time to extract fat, which compared with RL, the saturated fatty acid (SFA) of SL decreased, and the monounsaturated fatty acid (MUFA) increased, and the content of other fatty acids also changed accordingly; RL has a high content of SFA and MUFA; FO is rich in n-3 PUFA (polyunsaturated fatty acid) such as EPA (Eicosapentaenoic Acid) and DHA (Docosahexaenoic Acid), and SO is rich in n-6 PUFA. Previous studies suggested that different dietary lipids could impose different influences on the blood lipid profiles, which were probably caused by their specific fatty acid composition (Maki et al. 2018; Poli et al. 2008). Due to the differences in the composition of different oils, their digestive properties are also different. After digestion and absorption in the gastrointestinal tract, long-term intake has different effects on intestinal health.

Different types and contents of fats have different effects on the body's growth performance and intestinal microbes. At present, epidemiological studies mainly focus on the side effects of saturated fat and the positive effects of unsaturated fatty acids, and most studies have shown that high-fat diets can disrupt the intestinal microflora of the body (Zhang et al. 2012; Ghosh et al. 2013). Different effects of high-fat diets rich in different oils on lipid metabolism, oxidative stress was investigated, gut mirobiota and the high-fat diet exerted detrimental effects on the specific alterations in the gut microbiota, which increased the *Actinobacteria* and *Enterococcaceae* abundance, also decreased *Bacteroidetes, Proteobacteria, Lactobacillales* and *microbiota* diversity (Liu et al. 2020). Different fat types also can affect the intestines, studies have shown that diets with different fat sources will have a certain impact on the microflora of the cecal contents of piglets (Li et al. 2017). By comparing the effects of different types of fats on the growth performance and microecological effects of rats, it is found that different types of fatty acids have different effects on the physiological functions, digestive functions and intestinal flora of rats.

However, the existing studies have not taken into account the body's real needs for fat, and most of them are short-term studies based on high fat or disease models (Gauze-Gnagne et al. 2020; Adil et al. 2018). It is not yet clear whether different dietary fats have an effect on the normal physiological indicators, intestinal morphology and intestinal flora of rats within the reasonable intake range. Therefore, we studied the effects of SO, RL, SL and FO on the blood indicators, intestinal morphology and intestinal flora of rats, and explored the mechanism in order to guide oil consumption and processing provide a certain reference.

## Materials and methods

### Animals and diets

All experiments were carried out in compliance with the relevant guidelines and regulations of the Ethical Committee of Experimental Animal Center of Yangzhou University. A total of 48 4-week-old female Wistar rats were housed in a specific pathogen-free animal center (SCXK (Jiangsu) 2017–0007, Yangzhou, Jiangsu, China). The temperature (24.0 ± 0.5 °C) and relative humidity (60 ± 10%) were kept constant during the experiment, with a 12-h light cycle. Rats were fed a standard chow diet during a 1-week acclimation period. Then, animals were assigned to one of five diet groups (eight rats in each group), that is, fat-free (BC), stewed lard (SL), refined lard (RL), fish oil (FO) and soybean oil (SO) groups. RL, FO and SO were commercially obtained. SL is made by simmering pork belly for 120 min, removing the broth, chopped, freeze-dried, and then extracted according to the method of Folch (1957). It was placed in a reduced-pressure vacuum drying oven at 40 °C for 48 h to completely remove the remaining organic solvents, and vacuum packaged at − 20 °C until use. The composition of fatty acids in different oil were shown in Additional file 1: Table S1. The diets were prepared according to the AIN-93G diet formulation and the diet formulation was shown in Additional file 1: Table S2. During the experiment, the feed of each rat was refreshed every day to prevent oxidative deterioration of fat in the feed.

Observe the rat's diet rule to ensure that the daily diet is basically free of excess food, and provide a certain basis for the feeding amount of each experimental group during the formal experiment. Body weight and feed intake of rats were routinely recorded for calculating the average daily gain.

### Sample collection

Fresh feces were collected after rats were fed for 14 days. Normally, the animals excrete feces when they are hung by their tails. The fecal samples were immediately frozen in liquid nitrogen and then stored at − 80 °C until further analyses. At 6 weeks of feeding, the blood of rats were collected without fasting, the tubes stood at room temperature for 30 min and then were centrifuged at 3000 *g* for 30 min to pellet the blood cells. Serum samples were collected and stored at − 80 °C. At 6 weeks, all the rats were euthanized by cervical dislocation. The liver, kidney, heart and spleen were taken and weighed. Relative weights of the liver, kidney, heart and spleen tissues were calculated according to body weight. The duodenum and jejunum tissues of rats were soaked in 4% paraformaldehyde solution for fixation.

### Morphology of intestine

In this experiment, we used H-E staining jejunum and observed the tissue structure by microscope. We placed the slice on the stage, adjusted the eyepiece and objective lens, select the typical field of view, and use the XDS-3FL4 inverted fluorescence microscope with its own lens to obtain the slice photos. Randomly selected the obtained intestinal section photos, use Image-Pro Plus 6.0 image analysis software (Media Cybernetics, USA) to measure the length of intestinal villi and depth of crypts, randomly select intact intestinal villi in the field of view, and 10 measurements were taken as one group.

### Serum biochemical indicators

Serum biochemical indicators containing glucose(GLU), triglycerides (TG), total cholesterol (TC), high density lipoprotein cholesterol (HDL-C), and low density lipoprotein cholesterol (LDL-C) were determined using the commercially available kits from Nanjing Jiancheng Bioengineering Institute (Nanjing,China).

### 16S rRNA gene sequencing

Total genomic DNA in fecal samples was extracted using the QIAamp DNA Stool Mini Kit (Qiagen 51 504, Dusseldorf, Nordrhein-westfalen, Germany) according to the manufacturer’s instructions. The DNA was quantified by a Nanodrop® spectrophotometer (Nanodrop 2000, Thermo Fisher Scientific,Waltham, Massachusetts). Purified DNA was used to amplify the V4 region of 16S rRNA, which is associated with the lowest taxonomic assignment error rate. All DNA samples were kept at − 20 °C until sequencing. The V4–V5 hypervariable region of the 16S ribosomal RNA gene was selected for amplification from DNA samples. Polymerase chain reaction (PCR) was performed in triplicate. Amplicons were extracted from 2% agarose gels and purified using the AxyPrep DNA Gel extraction kit (Axygen Biosciences, Union City, California) according to the manufacturer’s instructions and quantified using QuantiFluor™ -ST (Promega, Madison, Wisconsin). The pooled DNA product was used to construct Illumina Pair-End library following Illumina’s genomic DNA library preparation procedure. Then the amplicon library was double-end sequenced (2 × 300 bp) on an Illumina MiSeq platform (San Diego, California) according to the standard protocols.

### Bioinformatics analysis

Raw fastq files were trimmed and chimeric sequences were identified and removed from all samples to reduce noise, and operational taxonomic units (OTUs) were clustered with ≥ 97% similarity cutoff using QIIME. Then community richness estimator (Chao and ACE), diversity indices (Shannon and Simpson), and Good’s coverage were calculated (Schloss et al. 2009). Duncan’s multiple comparison was applied to compare averages between any two groups. Differences were considered significant if p values were < 0.05. Principal coordinate analysis (PCoA) and clustering analysis were applied on the basis of the OTUs to offer an overview of the fecal microbial composition (Lozupone et al. 2011). Multivariate analysis of variance (MANOVA) was conducted to further confirm the observed differences. Linear discriminant analysis effect size (LEfSe) analysis (http://huttenhower.sph.harvard.edu/galaxy/) was carried out to discover biomarkers for fecal bacteria and to distinguish between biological conditions among different groups (Segata et al. 2011). The R (pheatmap package) and Cytoscape were applied to visualize the relationships through correlation heatmap and network diagrams respectively. The Illumina sequencing raw data have been successfully registered with the SRA database (Submission ID: SUB9765077; BioProject ID: PRJNA741340).

### Statistics analysis

All of the data were generated using SPSS 16.0 software and are represented here as mean ± standard deviation (SD). Significant differences among factors were analyzed by one-way analysis of variance (ANOVA). For pairwise comparisons, post hoc Tukey’s honest significant difference (HSD) test and Tamhane T2 test were used under equal variance and unequal variance conditions, respectively. *P* value < 0.05 was considered to be statistically significant.

## Results

### Physical characteristics

**Fig. 1:**
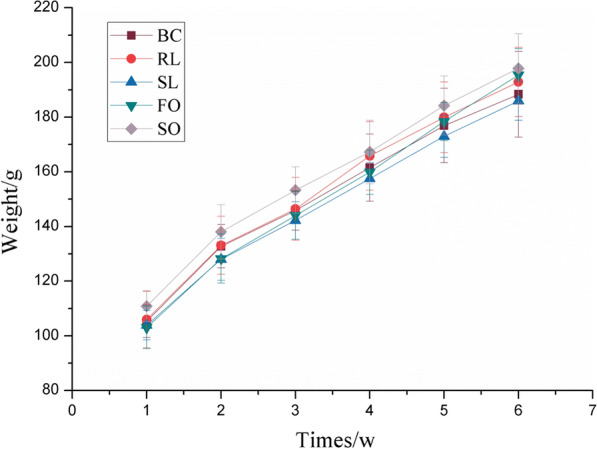
The body weight of rats fed with stewed lard, refined lard, fish oil and soybean oil during 6 weeks

During the experimental period, the rats in each group grew well, and there was no significant difference in initial body weight. It can be seen from Fig. 1 that the body weight of the rats has increased, and there is no significant difference between the groups at each time point (*P* > 0.05). The experimental results show that under the condition of intake of nutritional energy, the addition and type of dietary fat has no significant effect on the body mass of rats (*P* > 0.05). Under the conditions of intake and other nutritional energy, the addition and types of dietary fat have no significant effect on the body mass of rats.

It can be seen from Additional file 1: Table S3 that dietary fat has no significant effect on the heart index, spleen index and kidney index of rats in each group, indicating that the addition and type of dietary fat has no effect on rat kidney, heart and spleen. The liver index of RL group was significantly higher than that of rats in BC, FO and SO groups (P < 0.05).

### Blood biochemical indicators

As is shown in Table 1, compared with the RL group, the serum GLU content of rats in the SL group and the SO group was relatively low, but the difference was not significant. The fasting blood GLU value of rats in the FO group was lower than that of the BC group, and significantly lower than other dietary fat mixed diet groups (*P* < 0.05). Compared with the RL group, the serum TC content of the SL group was relatively lower. The serum TC content of rats in the FO group was significantly lower than that in the BC group and other dietary fat mixed diet groups (*P* < 0.05). Serum TG levels in BC and FO groups were significantly lower than in SL, RL and SO groups (*P* < 0.05). Compared with RL, SL can reduce the content of TC and TG in rat serum to a certain extent. The HDL-C of BC and SO groups was significantly higher than that of other groups (*P* < 0.05). The HDL-C content of FO group was lower than that of other groups. The level of LDL-C in the RL group was significantly higher than that in the FO and SO groups (*P* < 0.05). The serum HDL-C level of the SL group was higher than that of the RL group and the LDL-C level was lower than that of the RL group, but the difference was not significant (*P* > 0.05).

**Table 1:** Blood biochemical indices of the rats in response to different dietary fats (mmol/L)

Project	GLU	TC	TG	HDL-C	LDL-C
BC	4.44 ± 0.60^bc^	2.48 ± 0.27^b^	1.04 ± 0.08^b^	0.85 ± 0.18^a^	0.73 ± 0.08^ab^
SL	5.06 ± 0.51^ab^	2.81 ± 0.35^ab^	1.50 ± 0.26^a^	0.66 ± 0.09^b^	0.71 ± 0.17^abc^
RL	5.25 ± 0.43^a^	3.11 ± 0.39^a^	1.52 ± 0.29^a^	0.61 ± 0.12^b^	0.75 ± 0.09^a^
FO	4.07 ± 0.52^c^	1.97 ± 0.31^c^	1.21 ± 0.24^b^	0.59 ± 0.16^b^	0.60 ± 0.15^bc^
SO	4.77 ± 0.65^ab^	2.56 ± 0.34^b^	1.48 ± 0.30^a^	0.88 ± 0.18^a^	0.59 ± 0.07^c^

Different letters on the shoulder in the same column indicate significant difference in blood biochemical indices at the 0.05 significant level

### Morphology of rat intestine

As is shown in Table 2, the villus height of the homozygous diet supplemented with dietary fat was significantly higher than that of the BC group (*P* < 0.05). The villi and crypts in the intestinal tract of rats in the homozygous diet supplemented with dietary fat grew better, and their digestive physiological functions were better than those in the BC group, which helped the growth of animals. The intestinal wall thickness of the BC group was significantly lower than that of other homozygous diets supplemented with dietary fat (*P* < 0.05). The ratio of villus length/crypt depth in the BC group was significantly lower than that of other homozygous diets supplemented with dietary fat (*P* < 0.05). The ratio of villus height/crypt depth in the SL group was higher than that of the other groups, it shows that the intake of a certain amount of stewed lard can increase the villus height and crypt depth of the duodenum and jejunum, and enhance the digestion and absorption function of the intestine.

**Table 2:** Effect of dietary fat on the villus height, crypt depth and wall thickness of jejunum in rats

Project	BC	SL	RL	FO	SO
Villus height (μm)	821.76 ± 76.88^d^	1078.80 ± 43.50^ab^	1101.18 ± 55.01^a^	990.67 ± 103.25^bc^	925.78 ± 60.66^c^
Crypt depth (μm)	254.44 ± 13.68^d^	293.48 ± 10.12^ab^	303.94 ± 10.71^a^	271.42 ± 21.71^ cd^	279.48 ± 11.40^bc^
Wall thickness (μm)	202.80 ± 15.71^c^	277.36 ± 19.71^a^	279.55 ± 28.56^a^	242.86 ± 27.96^b^	256.15 ± 26.55^ab^
Villus height/crypt depth	3.23 ± 0.26^c^	3.68 ± 0.23^a^	3.62 ± 0.15^ab^	3.66 ± 0.36^a^	3.31 ± 0.18^bc^

Different letters on the shoulder in the same row indicate significant difference in morphological structure of jejunum at the 0.05 significant level

### Cholesterol and triglyceride content in rat liver

**Fig. 2:**
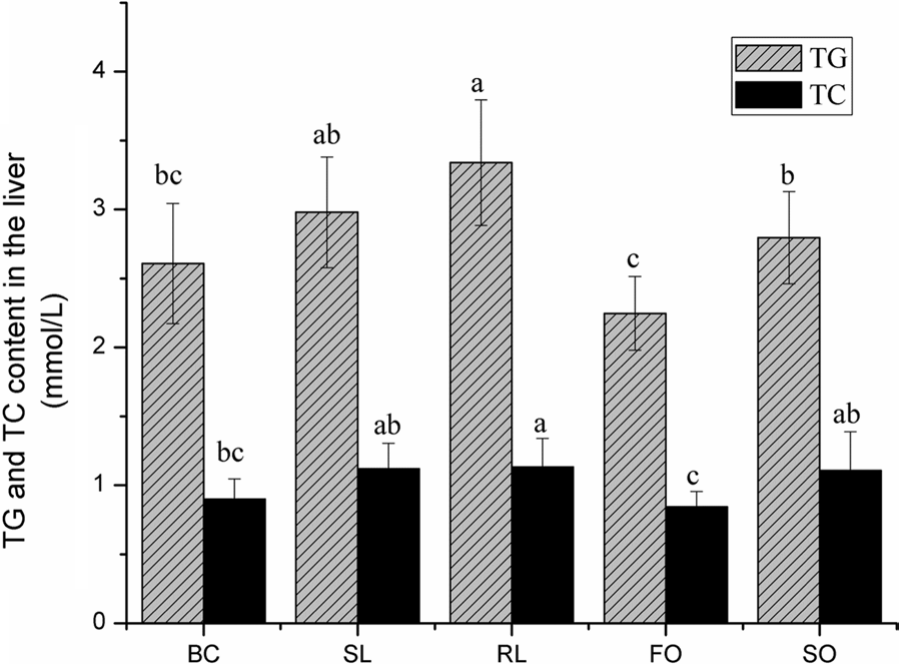
Content of cholesterol and triglyceride in rat liver. Different superscript letters indicate that the contents of TC and TG in liver are significantly different at 0.05 significant level

As is shown in Fig. 2, it is found that the TC and TG levels in the liver of the FO group were significantly lower than those of the other homozygous diets supplemented with dietary fat (*P* < 0.05). Fish oil not only significantly reduced the blood lipid content in rat serum, but also significantly reduced the cholesterol and triglyceride content in the liver.

### Richness and diversity analyses

The community richness estimators (Chao and ACE), and diversity indices (Shannon and Simpson) were calculated in order to evaluate the alpha diversity (Table 3). The Chao1 and ACE indexes of the SL group were higher than other experimental groups. Compared with other dietary fat mixed diet groups, the Chao1 and ACE indexes of the BC group were relatively low, and the Shannon index was relatively low. Experimental results show that SL can increase the abundance of intestinal flora to a certain extent, while fat-free diet will reduce the abundance and diversity of intestinal flora.

**Table 3:** Comparisons of gut bacteria on diversity index in response to dietary fats

Sample Name	Chao1	ACE	Shannon	Simpson
BC	1799.84 ± 407.96^a^	1867.58 ± 193.98^a^	8.32 ± 0.39^a^	0.98 ± 0.00^a^
SL	2051.61 ± 463.54^a^	2159.56 ± 735.43^a^	8.34 ± 0.31^a^	0.98 ± 0.01^a^
RL	1706.45 ± 156.80^a^	1858.77 ± 401.48^a^	8.45 ± 0.27^a^	0.99 ± 0.00^a^
FO	1937.37 ± 523.08^a^	2090.81 ± 432.75^a^	8.41 ± 0.30^a^	0.98 ± 0.01^a^
SO	1991.97 ± 590.71^a^	1940.17 ± 521.48^a^	8.18 ± 0.13^a^	0.98 ± 0.00^a^

Different letters on the shoulder in the same column indicate significant difference in diversity index at the 0.05 significant level

### Rat fecal microbe composition

**Fig. 3:**
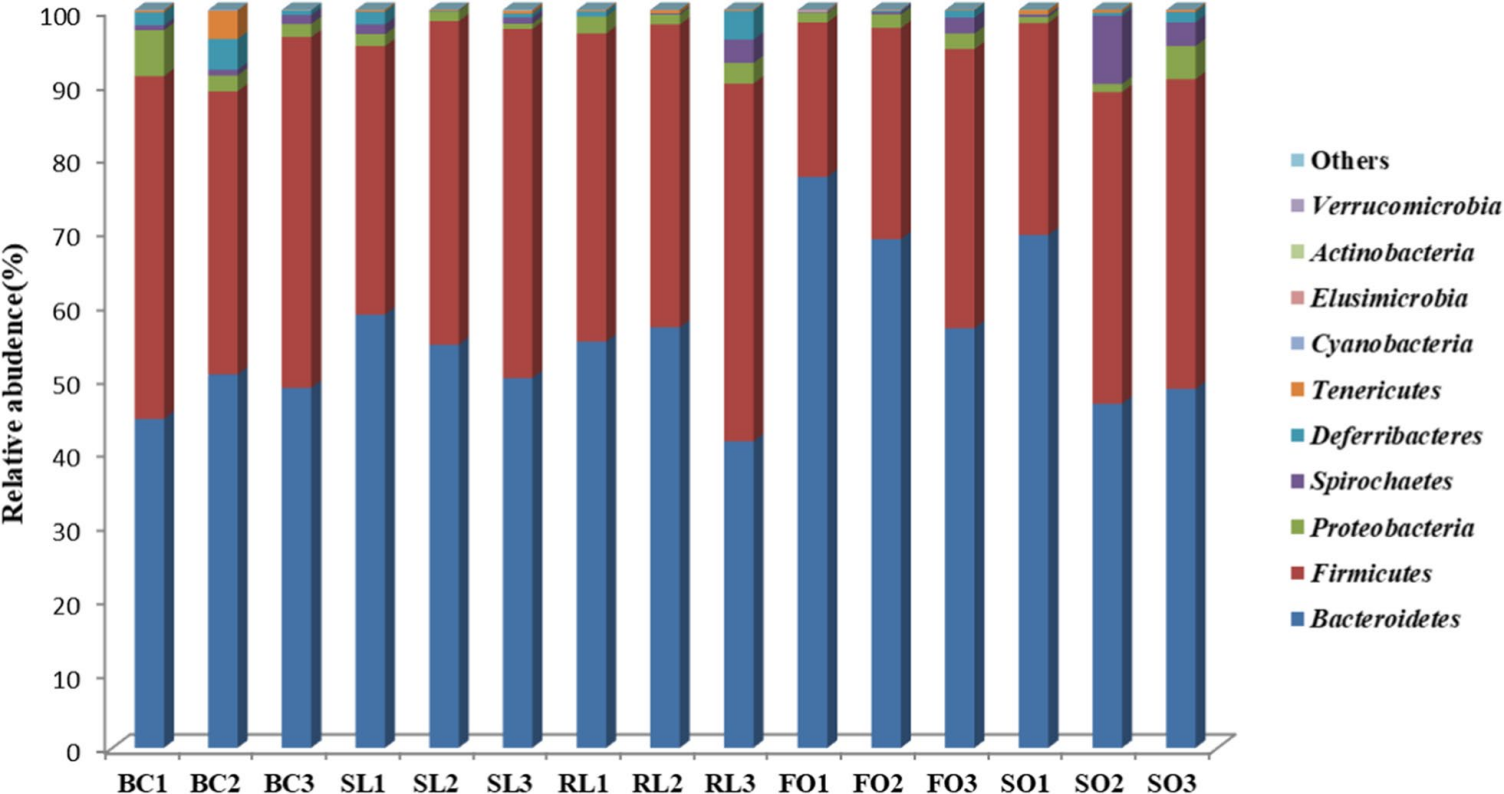
Effect of stewed lard, refined lard, fish oil and soybean oil on the phylum level of intestinal flora in rats

At the phylum level, *Bacteroidetes* and *Firmicutes* were predominant in all samples. In the BC group, *Bacteroidetes* was the most abundant phylum, accounting for 54.50%, followed by *Firmicutes* and *Proteobacteria* accounting for 42.47% and 1.22%, respectively. In the RL group, *Bacteroidetes* was the most abundant phylum, accounting for 51.20%, followed by *Firmicutes* and *Proteobacteria* accounting for 43.76% and 2.11%, respectively. In the FO group, *Bacteroidetes* was the most abundant phylum, accounting for 67.74%, followed by *Firmicutes* and *Proteobacteria*, accounting for 29.09% and 1.74%, respectively. In the SO group, *Bacteroidetes* was the most abundant phylum, accounting for 54.93%, followed by *Firmicutes* and *Proteobacteria* accounting for 37.65% and 2.11%, respectively. It can be seen from Fig. 3 that the dominant bacteria attached to the BC group, SL group, RL group, FO group and SO group are basically the same at the phylum level, and there are significant individual differences among all samples. The proportion of *Bacteroidetes* in each sample ranges from 41.52% to 77.38%, and the proportion of *Firmicutes* in each sample ranges from 20.90% to 48.52%. The proportion of *Proteobacteria* in each sample ranges from 0.71% to 6.20%. The ratio of *Firmicutes/Bacteroidetes* (F/B) in the SL group is 0.79 lower than that of the RL group. Compared with the fat-added diet group, the abundance of *Bacteroidetes* in the BC group decreased, and the abundance of *Firmicutes* increased. The F/B ratio of the BC group was the highest at 0.92. In addition, the abundance of *Bacteroidetes* in the FO group was significantly higher than that in the fat-free group (*P* < 0.05), the abundance of *Firmicutes* in the FO group was significantly lower than that in the BC, SL, and RL groups (*P* < 0.05), and the lowest F/B ratio in the FO group is 0.44.

**Fig. 4:**
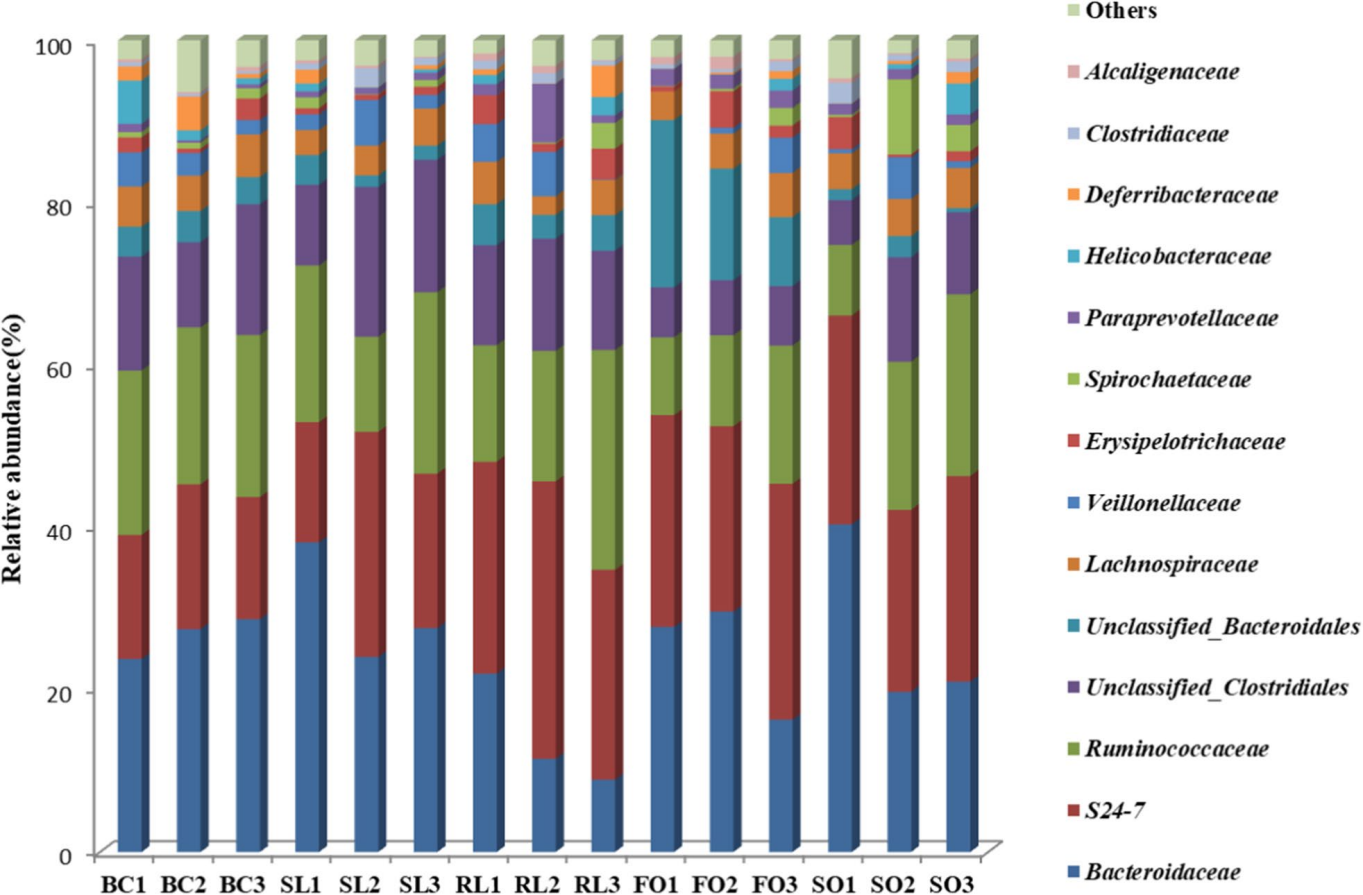
Effect of stewed lard, refined lard, fish oil and soybean oil on the family level of intestinal flora in rats

As is shown in Fig. 4, the abundance of *Bacteroidaceae* in the SL group was 29.95% higher than the other groups, and was significantly higher than that in the RL group (P < 0.05). There was no significant difference in the abundance of *Bacteroidaceae* in the BC, FO and SO groups. The abundance of *S24-7* in the BC group was lower than that of other dietary fat mixed diet groups, and significantly lower than that of the RL group (P < 0.05). The abundance of *S24-7* in SL group was not significantly different from BC, FO and SO groups. The highest abundance of *Ruminococcaceae* in the BC group was 19.82%, and the lowest abundance of *Ruminococcaceae* in the FO group was 12.60%.

## Discussion

The weight of rats in the fat-free group was lower than that in the homozygous diet group with added dietary fat, and the experimental results were similar to the results of previous studies. The liver is a key organ for the metabolism of various nutrients and lipid metabolism is inseparable from the liver. The synthetic function of the liver can combine fat with phosphoric acid or choline to form phospholipids, which are transported to other parts of the body. Once lipid metabolism disorder occurs, excess lipids are easy to accumulate in the liver and cause fatty liver disease (Kang et al. 2021). The liver index of the RL group was the highest, and was significantly higher than the FO and SO group. Therefore, RL has a higher probability of liver metabolism disorders or liver function damage.

Dietary fat has different effects on the serum indexes of rats. Compared with RL, SL can reduce the content of GLU, TC, TG, LDL-C in the serum to a certain extent, and increase the content of HDL-C. Fish oil is rich in n-3 PUFAs, especially EPA and DHA, which can improve the body's metabolic parameters (such as blood sugar, cholesterol and triglycerides) and reduce the accumulation of lipids in the carcass (Gondim et al. 2018). The atherosclerosis index of the RL group was significantly higher than that of the other groups, and the liver index was also higher than that of the other groups (Tung et al. 2019). The decrease in HDL-C levels in the FO group may be due to the richness of EPA and DHA in fish oil. As PUFA has no selective lipid-lowering effect, it reduces the level of HDL-C while reducing the TC level (Breetha and Ravichandra 2018). The serum TC content of rats fed FO was significantly lower than that of BC and other fat groups, which is consistent with related literature reports (Gondim et al. 2018; Harari et al. 2019). In contrast, RL tends to increase the level of LDL-C. Tung (2019) found that excessive intake of refined lard rich in SFA will increase serum TC, TG and LDL-C levels and reduce HDL-C levels, while intake of tea seed oil rich in MUFA will reduce blood lipid levels.

The gut is a complex and dynamic ecosystem. The composition of fecal microbiota is highly correlated with the colonic lumen and mucosa and moderately correlated with the distal small intestine (Yasuda et al. 2015). Small intestinal villi is the main tissue for nutrient absorption, and its height can reflect the mitotic activity of intestinal crypt cells. Studies have shown that changes in villi height can affect the surface area of intestinal villi, thereby affecting the absorption of nutrients (Czech et al. 2020; Caspary 1992). The increase in villus height helps to improve the intestinal health of rats, thereby increasing their digestion and absorption of nutrients ability. The depth of the crypt reflects the rate of intestinal villi forming epithelial cells, and the shallower crypt indicates that the maturation rate of intestinal epithelial cells has increased. Nutritional physiology studies have shown that the ratio of villi length/crypt depth can comprehensively reflect the physiological functions of the intestine. An increase in the ratio indicates an increase in the digestion and absorption function of the intestine, and a decrease in the ratio indicates a decline in the digestion and absorption function (Rehman et al. 2018). The villus height of the homozygous diet supplemented with dietary fat was significantly higher than that of the BC group, and the ratio of villus height/crypt depth in the SL group was higher than that of the other groups of rats, indicating that the consumption of stewed pork fat can enhance intestinal digestion and energy absorption. It can be seen that a fat-free diet can reduce fasting blood sugar and blood lipid levels to a certain extent, but it also reduces the intestinal nutrient absorption capacity. Compared with the RL group, SL can reduce the fasting blood sugar and blood lipid levels of the body to a certain extent, and can improve the intestinal nutrient absorption capacity.

Fish oil can significantly down-regulate the expression of fatty acid synthesis-related enzymes G6PDH, ME, FAS and other mRNA, and can increase the mRNA expression of fatty acid decomposing enzymes CPT-1 and CPT-2 in the rat liver, resulting in a decrease in liver and serum lipid concentrations(Godea et al. 2020). The content of TC and TG in the liver of the RL group was higher than that of the BC group and other homozygous diet groups with added dietary fat. Wang et al. (2013) compared the occurrence of obesity induced by high-fat diet with different oil components and found that the liver lipid content of the lard group was significantly higher than that of the soybean oil group. It is speculated that the type of fat is responsible for inducing lipid production and promoting lipid breakdown. The expression of related factors is related to the different utility. The results of this study show that compared with RL group, stewed lard has lower levels of TC and TG in the liver.

*Firmicutes* and *Bacteroides* are the most dominant bacterial phyla in the body, which can affect the energy extraction efficiency of the host and are related to the host's excessive obesity. Obese animals or humans have a higher abundance of *Firmicutes* in the gut flora (Kallus and Brandt 2012). In addition, many studies have found that obesity induced by high-fat diet will increase the ratio of *Firmicutes* to *Bacteroides*, which affects the metabolic function of intestinal microbes and increases their energy intake (Turnbaugh et al. 2006; Power et al. 2014; Kashyap et al. 2013). From the changes in the abundance of *Bacteroidetes* and *Firmicutes*, it can be proved that fish oil has a certain inhibitory effect on obesity and the beneficial effects of eating dietary fat on the intestinal flora, and refined lard is more likely to cause obesity than stewed lard.

The composition of the intestinal microbial colonies of five groups of rat fecal samples and the distribution and abundance of bacterial species were analyzed. The abundance of *Bacteroidaceae* in the SL group was higher than the other groups. The experimental results show that the SL can significantly increase the abundance of *Bacteroidaceae* in the body. The abundance of *Bacteroides* in the intestinal flora of obese rats induced by high-fat diet was lower than that in the normal group, green tea polyphenols can significantly increase the relative abundance of *Bacteroides* and Proteus after 3 weeks of intervention in rats, thereby reducing obesity in rats induced by high-fat diet (Guo et al. 2017).

*S24-7* under the phylum *Bacteroides* has not been isolated and cultured in vitro, and its biological characteristics are not yet clear. Ormerod et al. (2016) found that S24-7 is the main member of the gut microbiota and is highly present in the gastrointestinal tract of warm-blooded animals. They named it "Candidatus Homeothermaceae" and speculated that it belongs to Gram-negative anaerobes that can ferment carbohydrates extensively. Partial hepatectomy resulted in a significant increase in *S24-7* of the intestinal flora *Bacteroides* phylum and a significant decrease in *Firmicutes*, *Lachnospiraceae* and *Ruminococcaceae* (Liu et al. 2016).It is also suggested that liver metabolism and immune function are closely related to the abundance of *Lachnospiraceae*, *Ruminococcacea* and *S24-7*.The results show that fat-free diet will reduce the abundance of *S24-7* in the body to a certain extent. Rumen cocci mainly exist in the rumen, cecum and large intestine of mammals. Studies have shown that the rumen cocci in the intestine of high-fat rats will significantly decrease (Daniel et al. 2014), and the intake and types of dietary fat will have a certain impact on the relative abundance of *Ruminococcaceae* in the body. The abundance of *Lachnospiraceae*, *Desulfovibrionaceae*, *Acidaminococcaceae*, *Coriobacteriaceae* and *Bilophila* is positively correlated with obesity (Zhao et al. 2017).The experimental results showed that compared with the dietary fat group, the abundance of *Lachnospiraceae* in the BC group was relatively higher, and the abundance of *Lachnospiraceae* in the SL group was lower than that in the RL group. It shows that fat-free diet may also lead to obesity, refined lard is more likely to produce obesity than stewed lard.

Our study revealed that fat-free diet can reduce fasting blood sugar and blood lipid levels to a certain extent, but it also reduces the intestinal nutrient absorption capacity. Compared with the RL group, stewed lard can reduce the body's fasting blood sugar and blood lipid levels to a certain extent, and can improve the intestinal nutrient absorption capacity. Compared with other dietary fat groups, stewed pork fat will increase the abundance of *Bacteroidaceae* in rats, and fat-free diet will decrease the abundance of *S24-7* in rats. It shows that the addition and types of dietary fat will affect the distribution of rat intestinal flora to a certain extent. Therefore, we can try to choose some fats with higher unsaturated fatty acid content which similar to fish oil, and at the same time properly intake stewed lard to improve the abundance of intestinal flora.

## Supplementary Information

**Additional file 1:** **Table S1.** Fatty acid composition of experimental oils(g/100g oil). **Table S2.** Formula and calculated nutrient composition of experimental diet. **Table S3.** Effects of dietary fat on rat organ index. **Table S4.** Differences in *Bacteroidetes* and* Firmicutes *at the level of phylum in each group. **Table S5.** Differences in representative gut microbiota at the level of family in each group. **Figure S1.** H&E-stained of dietary fat intake for 6 weeks on the morphological structure of jejunum in rats.

## Data Availability

All data generated or analysed during this study are included in this published article.

## Abbreviations

BC: Blank control
SL: Stewed lard
RL: Refined lard
FO: Fish oil
SO: Soybean oil
Glu: Glucose
TC: Total cholesterol
TG: Total triglyceride
SFA: Saturated fatty acid
MUFA: Monounsaturated fatty acid
HDL-C: High-density lipoprotein
LDL-C: Low-density lipoprotein
F/B: The ratio of Firmicutes/Bacteroidetes
