# Supplementary material for: Effect of different types of oil intake on the blood index and the intestinal flora of rats
Source: AMB Express. 2022 May 5;12:49. doi: 10.1186/s13568-022-01387-w (PMC9072605; doi:10.1186/s13568-022-01387-w)
Supplement: Supplementary file 1 — Additional file 1: Table S1. Fatty acid composition of experimental oils(g/100g oil). Table S2. Formula and calculated nutrient composition of experimental diet. Table S3. Effects of dietary fat on rat organ index. Table S4. Differences in Bacteroidetes and Firmicutes at the level of phylum in each group. Table S5. Differences in representative gut microbiota at the level of family in each group. Figure S1. H&E-stained of dietary fat intake for 6 weeks on the morphological structure of jejunum in rats. [file 13568_2022_1387_MOESM1_ESM.docx]

**Supplementary information**

Effect of different types of oil intake on the blood index and the intestinal flora of rats

Yan Xu^1^, Wenzheng Zhu^1,2^, Qingfeng Ge^1^, Xiaoyan Zhou^1*^

^1^School of Food Science and Engineering, Yangzhou University, No.196 Huayang West Road, Hanjiang District, Yangzhou City, Jiangsu Province 225127, China;

^2^College of Animal Science and Technology, Yangzhou University, No.196 Huayang West Road, Hanjiang District, Yangzhou City, Jiangsu Province 225127, China;

Email Addresses: xuyanlp@yzu.edu.cn (YX); zhuwz@yzu.edu.cn(WZ); qfge@yzu.edu.cn(QG); yzuxyz@163.com(XZ)

*Correspondence: [yzuxyz@163.com(XZ)](mailto:yzuxyz@163.com(XZ))

**1.Fatty acid composition of different groups**

**Table S1 Fatty acid composition of experimental oils（g/100g oil）**

| Fatty acid | SL | RL | SO | FO |
| --- | --- | --- | --- | --- |
| C12:0 | 0.08 | 0.11 | 0.00 | 0.00 |
| C14:0 | 1.55 | 1.76 | 0.07 | 1.13 |
| C15:0 | 0.02 | 0.00 | 0.00 | 0.41 |
| C16:0 | 25.85 | 24.79 | 10.89 | 20.64 |
| C17:0 | 0.11 | 0.25 | 0.05 | 1.15 |
| C18:0 | 11.34 | 15.25 | 4.86 | 6.72 |
| C19:0 | 0.01 | 0.00 | 0.00 | 0.00 |
| C20:0 | 0.22 | 0.22 | 0.41 | 1.26 |
| C14:1 | 0.03 | 0.04 | 0.01 | 0.02 |
| C16:1 | 3.51 | 2.55 | 2.11 | 0.12 |
| C17:1 | 0.15 | 0.15 | 0.03 | 0.36 |
| C18:1 | 44.65 | 37.81 | 22.44 | 27.78 |
| C19:1 | 0.06 | 0.00 | 0.00 | 0.15 |
| C20:1 | 1.04 | 0.81 | 0.17 | 2.97 |
| C16:2 | 0.02 | 0.00 | 0.00 | 0.18 |
| C18:2 | 10.64 | 15.65 | 52.61 | 17.48 |
| C20:2 | 0.41 | 0.61 | 0.00 | 0.25 |
| C18:3 | 0.01 | 0.00 | 6.35 | 0.30 |
| C20:3 | 0.09 | 0.00 | 0.00 | 2.61 |
| C20:4 | 0.13 | 0.00 | 0.00 | 0.11 |
| C20:5 | 0.03 | 0.00 | 0.00 | 15.79 |
| C22:4 | 0.05 | 0.00 | 0.00 | 0.57 |
| C22:6 | 0.00 | 0.00 | 0.00 | 12.68 |
| SFA | 39.18 | 42.38 | 16.28 | 31.31 |
| MUFA | 49.44 | 41.36 | 24.76 | 31.40 |
| PUFA | 11.38 | 16.26 | 58.96 | 37.29 |
| UFA | 60.82 | 57.62 | 83.72 | 68.69 |
| UFA/SFA | 1.55 | 1.36 | 5.14 | 2.19 |

Note：SL, stewed lard group; RL, refined lard group ; FO, fish oil group; SO, soybean oil group.

**2.** **Feed composition**

**Table S2 Formula and calculated nutrient composition of experimental diet**

| Component (g/kg) | BC | SL | RL | FO | SO |
| --- | --- | --- | --- | --- | --- |
| Casein | 180.35 | 200.0 | 200.0 | 200.0 | 200.0 |
| Corn starch | 519.82 | 397.49 | 397.49 | 397.49 | 397.49 |
| Dextrin | 119.03 | 132.0 | 132.0 | 132.0 | 132.0 |
| Sucrose | 90.17 | 100.0 | 100.0 | 100.0 | 100.0 |
| Stewed lard | - | 70.0 | - | - | - |
| Refined lard | - | - | 70.0 | - | - |
| Fish oil | - | - | - | 70.0 | - |
| Soybean oil | - | - | - | - | 70.0 |
| Cellulose | 45.09 | 50.0 | 50.0 | 50.0 | 50.0 |
| Minerals | 31.56 | 35.0 | 35.0 | 35.0 | 35.0 |
| Vitamins | 9.02 | 10.0 | 10.0 | 10.0 | 10.0 |
| L-cystine | 2.71 | 3.0 | 3.0 | 3.0 | 3.0 |
| Choline Chloride | 2.25 | 2.5 | 2.5 | 2.5 | 2.5 |
| Tert-butyl hydrogen | 0.01 | 0.01 | 0.01 | 0.01 | 0.01 |
| Nutritional level |  |  |  |  |  |
| Total energy（Kcal/g） | 3.4 | 3.7 | 3.7 | 3.7 | 3.7 |
| Protein（%） | 19.4 | 19.4 | 19.4 | 19.4 | 19.4 |
| Carbohydrates（%） | 80.6 | 63.6 | 63.6 | 63.6 | 63.6 |
| Fat（%） | 0 | 16.9 | 16.9 | 16.9 | 16.9 |

Note：BC, fat-free group; SL, stewed lard group; RL, refined lard group ; FO, fish oil group; SO, soybean oil group.

**3. Organ index**

**Table S3 Effects of dietary fat on rat organ index**

| Project | heart | liver | spleen | kidney |
| --- | --- | --- | --- | --- |
| BC | 0.37±0.03^a^ | 2.53±0.09^bc^ | 0.20±0.01^a^ | 0.63±0.03^a^ |
| SL | 0.35±0.03^a^ | 2.58±0.06^ab^ | 0.21±0.02^a^ | 0.64±0.06^a^ |
| RL | 0.38±0.04^a^ | 2.68±0.12^a^ | 0.20±0.01^a^ | 0.67±0.04^a^ |
| FO | 0.35±0.03^a^ | 2.45±0.09^c^ | 0.21±0.01^a^ | 0.65±0.05^a^ |
| SO | 0.37±0.05^a^ | 2.57±0.12^bc^ | 0.21±0.02^a^ | 0.65±0.02^a^ |

Note：BC, fat-free group; SL, stewed lard group; RL, refined lard group ; FO, fish oil group; SO, soybean oil group. Different letters on the shoulder in the same column indicate significant difference in organ index at the 0.05 significant level.

**4. Intestinal flora at phylum level**

**Table S4 Differences in *Bacteroidetes* and *Firmicutes* at the level of phylum in each group**

| Sample Name | *Bacteroidetes*（%） | *Firmicutes*（%） | *Firmicutes / Bacteroidetes* |
| --- | --- | --- | --- |
| BC | 48.00±3.09^b^ | 44.11±4.99^a^ | 0.92±0.14^a^ |
| SL | 54.50±4.31^ab^ | 42.48±5.59^a^ | 0.79±0.16^ab^ |
| RL | 51.20±8.42^ab^ | 43.76±4.14^a^ | 0.88±0.24^a^ |
| FO | 67.74±10.29^a^ | 29.09±8.44^b^ | 0.44±0.19^b^ |
| SO | 54.93±12.66^ab^ | 37.65±7.74^ab^ | 0.73±0.27^ab^ |

*Different letters on the shoulder in the same column indicate significant difference in the level of phylum at the 0.05 significant level.*

**5. Intestinal flora at family level**

**Table S5 Differences in representative gut microbiota at the level of family in each group**

| Sample Name | *Bacteroidaceae*（%） | *S24-7*（%） | *Ruminococcaceae*（%） | *Lachnospiraceae*（%） |
| --- | --- | --- | --- | --- |
| BC | 26.67±2.55^ab^ | 16.06±1.54^c^ | 19.82±0.47^a^ | 4.85±0.47^a^ |
| SL | 29.95±7.34^a^ | 20.49±6.58^bc^ | 17.79±5.45^a^ | 3.77±0.74^a^ |
| RL | 14.14±6.90^b^ | 28.72±4.74^a^ | 19.15±6.91^a^ | 3.93±1.50^a^ |
| FO | 24.57±7.21^ab^ | 25.98±3.13^ab^ | 12.60±3.89^a^ | 4.42±0.98^a^ |
| SO | 27.05±11.59^ab^ | 24.50±1.78^ab^ | 16.43±6.99^a^ | 4.65±0.27^a^ |

*Different letters on the shoulder in the same column indicate significant difference in the level of family at the 0.05 significant level.*

**6. Intestinal biopsy**

**
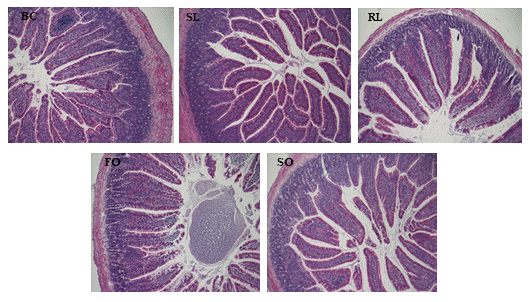
**

**Figure S1 H&E-stained of dietary fat intake for 6 weeks on the morphological structure of jejunum in rats**
